# The blue light signal transduction module FaCRY1-FaCOP1-FaHY5 regulates anthocyanin accumulation in cultivated strawberry

**DOI:** 10.3389/fpls.2023.1144273

**Published:** 2023-06-09

**Authors:** Yongqiang Liu, Li Tang, Yiping Wang, Lianxi Zhang, Shiqiong Xu, Xiao Wang, Wen He, Yunting Zhang, Yuanxiu Lin, Yan Wang, Mengyao Li, Xiaorong Wang, Yong Zhang, Ya Luo, Qing Chen, Haoru Tang

**Affiliations:** ^1^ College of Horticulture, Sichuan Agricultural University, Chengdu, China; ^2^ Institute of Pomology and Olericulture, Sichuan Agricultural University, Chengdu, China

**Keywords:** strawberry, anthocyanin, blue light, HY5, COP1, BBX protein

## Abstract

Anthocyanins have important physiological functions and are beneficial to the improvement of fruit quality in strawberry. Light is important for anthocyanin biosynthesis, and specific light quality was identified to promote anthocyanin accumulation in many fruits. However, research on the molecular mechanisms of anthocyanin accumulation regulated by light quality in strawberry remains limited. Here we described the effects of red- and blue-light irradiation on anthocyanin accumulation in strawberry. The results showed that blue light, rather than red light, could lead to the rapid accumulation of anthocyanins after exposure to light for 48 hours. The transcriptional levels of anthocyanin structural and regulatory genes displayed similar trend to the anthocyanin content. To investigate the mechanism of blue light-induced anthocyanin accumulation, the homologs of Arabidopsis blue light signal transduction components, including the blue light photoreceptor *FaCRY1*, an E3 ubiquitin ligase *FaCOP1* and light-responsive factor *FaHY5*, were cloned from the strawberry cultivar ‘Benihoppe’. The protein-protein interaction of FaCRY1-FaCOP1-FaHY5 was revealed by yeast two-hybrid and fluorescence signal assays. Functional complementation analysis showed that overexpression of either *FaCOP1* or *FaHY5* restored the anthocyanin content and hypocotyl length in corresponding Arabidopsis mutants under blue light. Moreover, dual-luciferase assays showed that FaHY5 could increase the activity of *FaRAP* (anthocyanin transport gene) promoter and that this function relied on other, likely B-box protein FaBBX22, factors. The overexpression of *FaHY5*-*VP16* (chimeric activator form of FaHY5) and *FaBBX22* promoted the accumulation of anthocyanins in transgenic strawberry plants. Further, transcriptomic profiling indicated that the genes involved in the phenylpropanoid biosynthesis pathway were enriched in both *FaHY5*-*VP16-*OX and *FaBBX22*-OX strawberry plants. In summary, our findings provide insights into a mechanism involving the regulation of blue light-induced anthocyanin accumulation *via* a FaCRY1-FaCOP1-FaHY5 signal transduction module in strawberry.

## 1 Introduction

Cultivated strawberry (*Fragaria* × *ananassa*) is a highly popular fleshy fruit, widely cultivated for its distinctive taste, abundance of nutrients, and high economic value. The bright red color of ripened strawberry is due to the accumulation of anthocyanins. In addition to giving fruits a showy appearance, anthocyanins are highly attractive to consumers due to their antioxidant activities (Skrovankova et al., 2015). Anthocyanins accumulate specifically in various plant tissues for different functions. For example, anthocyanins in the flowers and fruits provide visual cues to attract pollinators and seed dispersers, while the anthocyanins in vegetative organs act as antioxidants to increase plant resistance to biotic and abiotic stresses (Jaakola, 2013).

**Figure 1:**
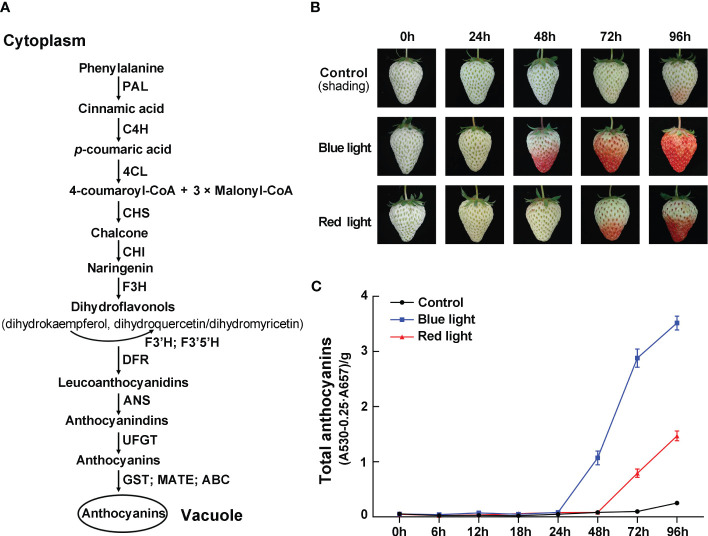
The effects of red and blue light irradiation on the color of strawberry fruits. **(A)** Schematic diagram of anthocyanin biosynthesis pathway. **(B)** The color changes of ‘Benihoppe’ strawberry during the light irradiation treatments. **(C)** The total anthocyanin content at different time points. Data are presented as means ± standard deviations (n=3).

Anthocyanins are water-soluble pigments derived from the flavonoid pathway. The process of anthocyanin biosynthesis is mainly composed of multi-step enzymatic reactions (**Figure 1A**) and has been well established in plants (Zhao et al., 2022). Generally, phenylalanine is considered as the precursor for anthocyanin biosynthesis, catalyzed by phenylalanine ammonia-lyase (PAL), cinnamate 4-hydroxylase (C4H), and 4-coumarate: CoA ligase (4CL) to produce 4-coumaroyl-CoA. Then, one molecule 4-coumaroyl-CoA and three molecules of malonyl-CoA are catalyzed by chalcone synthase (CHS), chalcone isomerase (CHI), and flavonoid 3-hydroxylase (F3H) to produce dihydrokaempferol. Dihydrokaempferol can be further hydroxylated by flavonoid 3’-hydroxylase (F3’H) and flavonoid 3’,5’-hydroxylase (F3’5’H) into other dihydroflavonols. The structural genes involved in encoding enzymes in these reactions are called early biosynthesis genes (EBGs). Next, the late biosynthesis genes (LBGs) encode a series of enzymes, including dihydroflavonol 4-reductase (DFR), anthocyanidin synthase (ANS), and several glycosyltransferases (UFGTs), which have broad substrate specificity to convert dihydroflavonols to different colored anthocyanins. At the final step, some transporters, such as glutathione S-transferases (GSTs), multidrug and toxic extrusion (MATE), and ATP-binding cassette (ABC) proteins, transfer anthocyanins from the endoplasmic reticulum to the vacuole for storage (Zhao and Dixon, 2010). The absence of anthocyanin-transporting GST often led to dramatic changes in anthocyanin content, like *TT19* (transparent testa 19) in Arabidopsis, *MdGSTF6* in apple, *PpGST1* in peach, and *RAP* (reduced anthocyanins in petioles) in strawberry (Kitamura et al., 2004; Luo et al., 2018; Jiang et al., 2019; Zhao et al., 2020). Transcription factors, such as MYB, bHLH, and WD40, form the MBW ternary complexes, which bind to the promoter of structural genes and finely regulate the transcription of these genes (Xu et al., 2015). The R2R3 MYB TFs had been identified as the major regulators in regulating anthocyanin biosynthesis by the MBW complexes in various plant species (Allan and Espley, 2018). For example, the activator-type R2R3 MYB10 TF is a well-known determinant of anthocyanin biosynthesis during fruit ripening in strawberry, while repressor-type R2R3 MYB1 TF plays a negative role by down-regulating the expression of LBGs in anthocyanin biosynthesis pathway (Aharoni et al., 2001; Paolocci et al., 2011; Lin-Wang et al., 2014; Castillejo et al., 2020). In MBW complexes, bHLH TFs regulate anthocyanin production mainly through interaction with MYB proteins, and WD40-repeat TFs do not directly bind to the promoter of target genes, which can provide a stable platform for MYB and bHLH to form the MBW complexes (Xu et al., 2015).

In addition to genetic factors, environmental factors such as light, drought, and temperature can also affect anthocyanin biosynthesis (Saigo et al., 2020). Among various factors, light plays an integral role, both as the energy source for photosynthesis and as a signaling factor that triggers plants to produce anthocyanins in response to changes in the light environment. Light-induced anthocyanin biosynthesis is mainly achieved by the regulation of light-responsive TFs, which can directly control the expression of anthocyanin structural genes (Ma et al., 2021). At present, many TFs have been found to be involved in light-induced anthocyanins in horticultural plants, such as MdMYB1 and MdMYB10 in *Malus domestica*, PybZIPa in *Pyrus pyrifolia*, FvbHLH9 in *Fragaria vesca*, DcTTG1 in *Dendrobium candidum* (Takos et al., 2006; Espley et al., 2007; Liu et al., 2019; Li et al., 2020; Jia et al., 2021). Under darkness, some light-responsive TFs are degraded by E3 ubiquitin ligase complex *via* 26S proteasome pathway, thereby leading to the deactivation of light signal transduction (Hoecker, 2017). CONSTITUTIVE PHOTOMORPHOGENIC 1 (COP1), a RING-finger E3 ligase, is well-known as a molecular switch in light-regulated plant growth and development (Podolec and Ulm, 2018). In Arabidopsis, COP1 interacts with the positive regulators of light-induced anthocyanin biosynthesis, such as bZIP TF (ELONGATED HYPOCOTYL5, HY5), B-box TF (BBX21), MYB TF (PRODUCTION OF ANTHOCYANIN PIGMENT1, PAP1), to promote their ubiquitination and degradation in the nucleus (Osterlund et al., 2000; Maier et al., 2013; Xu et al., 2016). Upon light exposure, COP1 is excluded from the nucleus to the cytosol and its activity is reduced by the light-activated photoreceptors (Pacin et al., 2014; Podolec and Ulm, 2018). For instance, Arabidopsis photoreceptor cryptochromes (CRYs) interact with COP1 only after seedlings are exposed to blue light, which disrupts the COP1-mediated ubiquitin complex and increases the protein stability of COP1-targeted substrates (Holtkotte et al., 2017). Among COP1-targeted substrates, HY5 is believed to be one of the central role in light signaling as a modulator of hypocotyl growth and anthocyanin accumulation (Gangappa and Botto, 2016). It has been shown that HY5 increases the transcriptional level of anthocyanin-related genes by directly binding to *cis*-regulatory elements (Lee et al., 2007; Zhang et al., 2011). Furthermore, *in vitro* and *vivo* analyses showed that HY5 lacks the transactivation domain, and it generally acts as a DNA-binding transcriptional regulator and relies on the cofactors to provide transcriptional activity (Ang et al., 1998; Burko et al., 2020). Recently, some light-responsive B-box TFs, such as AtBBX20/21/22 in Arabidopsis, MdBBX20 in apple, PpBBX16 in pear, have been identified as the interacting proteins of HY5 (Bai et al., 2019a; Fang et al., 2019; Bursch et al., 2020). The HY5/BBXs transcription complexes promote light-induced anthocyanin accumulation by regulating structural and regulatory genes (Bai et al., 2019b).

Previous studies have shown that the effects of light on anthocyanin accumulation vary with different wavelengths. Different light quality treatments were applied to red pear 150 days after flowering. After 72 hours of exposure, blue light was found to promote the accumulation of anthocyanins in the pear, while red light had little effect (Tao et al., 2018). Samkumar et al. (2021) found that both red and blue light increased the anthocyanin content of bilberry (*Vaccinium myrtillus* L.) after 6 days of continuous light treatment during fruit ripening. In apple, the expression of anthocyanin-related genes responded to UV-B irradiation, and the content of anthocyanin increased with the prolonged exposure time to UV-B irradiation (Fang et al., 2019). Our previous works found that pelargonidin 3-glucoside is the most abundant anthocyanin in the fruits of ‘Benihoppe’ strawberry, and its biosynthesis was promoted by blue light, but the related mechanism remains unclear (Zhang et al., 2018). To clarify the effects of different light qualities on the fruit coloration of strawberry, the detached fruits at white fruit stage were treated to continuous red- and blue-light irradiation for 96 hours. The results showed that blue light treatment accelerates the accumulation of anthocyanins. Then, the protein-protein interactions of blue light signal transduction components (FaCRY1, FaCOP1, and FaHY5) were confirmed. We further characterized the role of FaHY5 and provided molecular evidences that FaHY5 requires the participation of cofactors to promote transcription in anthocyanin accumulation. Our findings confirmed that blue light-induced anthocyanin accumulation is regulated by a conserved signal transduction pathway in strawberry.

## 2 Materials and methods

### 2.1 Plant materials and growth conditions

An octoploid strawberry cultivar ‘Benihoppe’ with red fruit flesh was used in this study. The strawberry was grown in a transparent plastic greenhouse of the Sichuan Agricultural University. Before light quality treatments, the strawberry fruits at the stage of big green fruit were covered with double-layered yellow-and-black paper bags for light-proofing until the fruit developed to the white fruit stage (approximately 7 days). Select bagged fruits (white fruit stage) of similar size for harvesting. A plant growth cabinet (ZDN-400-2, Dongnan Instrument Co., Ltd., Ningbo, China) consisting of a red LED (peak wavelength 660 nm) and a blue LED (peak wavelength 450 nm) was used for light quality treatment. Wrap the stalks of strawberry fruits with moistened cotton wool and place the fruits in a clear crisper. All fruits were transferred into the plant growth cabinet (10 °C, relative humidity 70%), and maintained in the dark for 2 days. For the detached fruits light quality treatments, the fruits were randomly divided into three groups. One group was irradiated with blue light, and another group was irradiated with red light, and the third group was kept in the dark as a control group. Samples were collected at 0, 6, 12, 18, 24, 48, 72 and 96 h after irradiation. Six individual fruits were used for each time point, and which were replicated independently three times. All simple were stored at -80°C after immediately freezing in liquid nitrogen.

The Arabidopsi*s hy5-1* mutant and *cop1-6* mutant were purchased from https://www.arabidopsis.org/.

### 2.2 Anthocyanin extraction and measurement

The total anthocyanin content of strawberry and Arabidopsis were assayed by using the method as described in our previous study (Liu et al., 2022). The fresh tissues were ground into a powder and subsequently soaked in an extraction solution (70% methanol, 27% water, 2% formic acid, and 1% trifluoroacetic acid) by mixing 0.1 g of the powder with 1 mL of the solution at 4°C in the dark. After 12 h, the mixture was centrifuged at 13,000 rpm for 10 min at 4°C. Then, the supernatant was collected for absorbance measurement. The absorbance at 530 nm and 657 nm was measured using a UV spectrophotometer (MAPADA, China). The total anthocyanin content was calculated using the following formula: Total anthocyanins = [A530 - (0.25 × A657)]/M, where A530 and A657 are the absorbance values at the respective wavelengths and M is the weight of the fresh tissue (in grams).

### 2.3 Quantitative real-time PCR

The relative expression levels of target genes were detected by real-time quantitative reverse transcription PCR (RT-qPCR) technology. Total RNA of strawberry and Arabidopsis were extracted from the frozen tissues using the method as described in the previous study (Chen et al., 2012). Then, the integrity of the RNA was verified with NanoDrop™ One spectrophotometer (Thermo Scientific). A 1 μg sample of RNA were reversely transcribed into complementary DNA (cDNA) by using RT Easy™ II cDNA Synthesis Kit (Foregene, China). The RT-qPCR were performed with the Bio-Rad CFX96 system (Bio-Rad, USA) using Real Time PCR Easy™ SYBR Mix (Foregene, China). The relative fold differences of target genes were calculated using the 2^-ΔΔCT^ method. All primers used for RT-qPCR are listed in **Supplementary Table 1**.

### 2.4 Arabidopsis transformation and phenotype analysis

The Arabidopsis mutant line *hy5*-*1* is in the Landsberg *erecta* (L*er*) ecotype and *cop1-6* is in the Columbia-0 (Col-0) ecotype, which were used for the functional complementation assays. The full-length CDS of *FaHY5* and *FaCOP1* were isolated from the cDNA of ‘Benihoppe’ strawberry and cloned into the modified pCAMBIA1301 expression vector containing a CaMV 35S (35S) promoter for overexpression. The recombinant plasmid was transformed into *A*. *tumefaciens* strain GV3101. Genetic transformation was conducted according to the floral dip method (Clough and Bent, 1998). The transgenic Arabidopsis lines were selected on MS medium containing 50 mg/L hygromycin B. For each type of transgenic Arabidopsis, three lines with the relatively high expression levels of target gene were selected for measurements of hypocotyl length and anthocyanin content.

The T3 seeds were grown into seedlings in a growth cabinet with blue light (100 μmol·m^-2^·s^-1^), 8-hour dark/16-hour light at 23°C. After 10 days, the hypocotyl length and anthocyanin content of ≥ 20 Arabidopsis seedlings were measured.

### 2.5 Yeast two-hybrid assay

In Arabidopsis, previous works have shown that the C-terminal domain of AtCRY1 is an important region for the interaction between AtCRY1 and AtCOP1 through yeast two-hybrid (Y2H) assay (Wang et al., 2001; Yang et al., 2001). Further, Holtkotte et al. (2017) found that blue light enhanced the interaction intensity of AtCRY1 protein and AtCOP1 protein in yeast. To investigate the protein-protein interaction between FaCRY1 and FaCOP1, the Y2H assays were conducted as described by our previous study (Liu et al., 2022). The C terminal region (amino acids 479-673) of FaCRY1 was amplified and inserted into multiple cloning site (MCS) of bait vector pGBKT7 (BD). The full-length CDS of FaCOP1 was inserted into the MCS of prey vector pGADT7 (AD). The bait and prey constructs were co-transformed into yeast strain Y2HGold using the PEG/LiAc-based method. Following transformation, the yeast cells were spread onto synthetically defined medium (SD/-Trp-Leu) and incubated at 30 °C for 3 days. Then, 10 independent clones were selected and transferred to SD/-Trp-Leu-Ade-His medium supplemented with Aureobasidin A (100 ng/mL) and X-α-gal and cultured under blue light (50 μmol·m^-2^·s^-1^) for 4 days. All primers used for the vector construction are listed in **Supplementary Table 1**.

### 2.6 Fluorescence and confocal microscopy

The localization assay of FaCOP1 and FaHY5 were taken in *Nicotiana benthamiana* mesophyll cells. The FaCOP1-eGFP fusion construct was produced by inserting the CDS of FaCOP1 without termination codon into the pYTSL-16 vector, which has a 35S-MCS-terminator expression cassette. The eGFP of pYTSL-16 was replaced by red fluorescent protein (mCherry), which was fused with FaHY5 to form 35S:FaHY5-mCherry recombinant plasmid (FaHY5-mCherry). The FaCOP1-eGFP and FaHY5-mCherry were co-expressed in tobacco leaves *via* an *Agrobacterium*-mediated method (Ye et al., 2021). Fluorescence images were taken with a 20 × objective on a confocal laser scanning microscope (FV3000, OLYMPUS, Japan).

### 2.7 Generation of transgenic strawberry

For stably expressing the activator form of FaHY5 in strawberry, the herpes simplex virus VP16 transactivation domain was fused to the C-terminus of FaHY5 (named *FaHY5*-*VP16*) by gene synthesis (Tsingke, Chengdu, China) and the product was subcloned into pCAMBIA1301 plant expression vector, which contains a 35S-MCS-NOS expression cassette. The recombinant plasmid for *FaBBX22*-overexpression was obtained in our previous work (Liu et al., 2022). All constructs were transformed into *A*. *tumefaciens* strain GV3101 for genetic transformation of ‘Benihoppe’ strawberry, using a method previously described with minor modifications (Liu et al., 2022). Briefly, positive transgenic calli were identified by using β-glucuronidase (GUS) histochemical staining and cultured on selective medium, which were transferred into the new selective medium every 20 days until the regenerated shoots appeared. The new shoots were transferred into the 0.5 × MS (1/2 microelements) medium with 5 mg/L hygromycin B for rooting. After 1 month, the regenerated plants were transferred into subculture medium (1 × MS, 0.5 mg/L 6-benzylaminopurine, 0.1 mg/L indole-3-butyric acid, 50 mg/L carbenicillin and 50 mg/L timentin, 2.5 mg/L hygromycin B, 3% sucrose, 0.7% agar, pH 5.8) for propagation. The specific primer for the hygromycin resistance gene (*HygR*) was adopted to detect positive transgenic plants. Subsequently, RT-qPCR was performed to assess the relative expression level of the target genes (*FaHY5* or *FaBBX22*) in different transgenic lines, and at least three independent transgenic lines for each construct were obtained.

### 2.8 RNA-seq analysis

The transgenic strawberry lines with the highest expression level of target gene were selected for RNA-seq analysis, and the wild-type ‘Benihoppe’ strawberry was selected as control. All strawberry seedlings were grown in the dark at 23 °C for 3 days and then exposed to white light (100 μmol·m^-2^·s^-1^). After 12 hours of light exposure, the samples of at least 6 strawberry seedlings were collected for each replicate and snap-frozen in liquid nitrogen. The total RNAs were extracted as described above. 1 μg of total RNA was used to prepare RNA-seq transcriptome libraries using the TruSeqTM RNA sample preparation kit from Illumina (San Diego, CA). The libraries were sequenced (150 bp pair end) by Illumina NovaSeq 6000 sequencing (BIOZERON, Shanghai, China). The raw paired end reads were trimmed, and quality controlled by Trimmomatic. Then clean reads in each library were separately mapped against the *F*. ananassa genome with the v1.0.a2 annotation using hisat2 (https://ccb.jhu.edu/software/hisat2/index.shtml) software (Liu et al., 2021). The expression level for each gene was calculated using the fragments per kilobase of exon per million mapped reads (FPKM) method. R statistical package edgeR was used for differential expression analysis (http://www.bioconductor.org/packages/release/bioc/html/edgeR.html/). The genes with the logarithmic of fold change was greater than 2 and the false discovery rate (FDR) be less than 0.05 were considered as significantly DE. The KEGG pathway analysis was performed using KOBAS (http://kobas.cbi.pku.edu.cn/home.do).

### 2.9 Dual-luciferase assay

Our previous work showed that the anthocyanin transporter gene *FaRAP* is a potential target gene of FaHY5 (Liu et al., 2022). To examine the mechanism of FaHY5 in the transcriptional regulation of target gene, a dual-luciferase assay was conducted. The full-length CDS of *FaHY5*, *FaHY5*-*VP16* and *FaBBX22* were recombined into pGreenII 62-SK vector, respectively, named as effector constructs. The reporter construct was generated by cloning the promoter of *FaRAP*, which spanned 2000 bp upstream of the initiation codon, into the MCS of pGreenII 0800-LUC. *N*. *benthamiana* leaves were co-infiltrated with *A*. *tumefaciens* strain GV3101 (pSoup-p19) containing the reported effector and reporter construct (Hellens et al., 2005). After 48 hours of infiltration, 0.5 cm leaf discs were harvested, and the firefly luciferase/renilla luciferase (LUC/REN) activities were quantified using the dual-luciferase reporter assay system (Promega, USA) with Varioskan™ LUX (Thermo Scientific). Each sample was analyzed in at least four biological replicates.

### 2.10 Statistical analysis

The data were presented as the mean ± standard deviations (SDs). The statistical significance of differences was analyzed using Student’s *t*-test (** *p* < 0.01) and one-way analysis of variance (ANOVA) of SPSS Statistics software (SPSS 27.0, IBM, Chicago, IL, USA). Graphs were plotted using GraphPad Prism 8 (GraphPad software).

## 3 Results

### 3.1 Blue light rapidly induced anthocyanin accumulation in strawberry fruit

To investigate the effects of different light quality treatments on anthocyanin accumulation in strawberry fruit, the detached fruits were collected at the white fruit stage and treated with continuous blue light and red light. By visual inspection, the control group fruits kept in darkness remained bright green for the first 72 hours and started showing a slight reddish hue after 96 hours (**Figure 1B**). Under red light irradiation, there was no significant accumulation of anthocyanins within the first 48 hours of treatment, and anthocyanin accumulated gradually with the lengthening of treatment time. In contrast, blue-light treated fruits showed the fastest coloring speed, and these fruits turned noticeably red after 48 hours of light exposure (**Figure 1B**). Meanwhile, the total anthocyanin content in strawberry fruits were measured after 0, 6, 12, 18, 24, 48, 72 and 96 h of light exposure. At 48 hours of treatment, the total anthocyanin content increased sharply to 0.97 in blue light irradiation, while the anthocyanin was almost undetectable in red light irradiation and control group. During the treatment period, the total anthocyanin content of fruits was the highest at 96 h after blue light irradiation, which was 2.4-fold and 14-fold higher than that of red light irradiation and control group, respectively (**Figure 1C**). The results showed that the anthocyanin content was rapidly increased by blue-light exposure, which was consistent with visual appearance.

**Figure 2:**
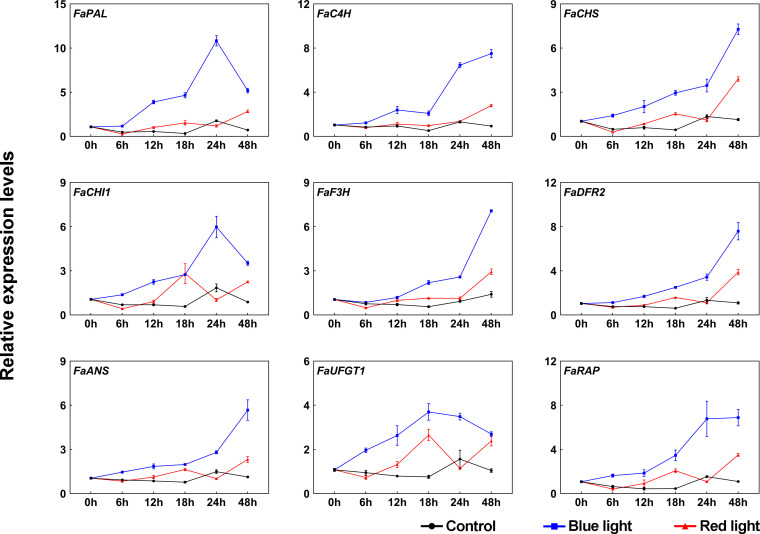
The effects of red and blue light irradiation on the transcriptional levels of anthocyanin structural genes. All dates were normalized to the transcriptional levels of *FaActin* gene (accession: AB116565.1). Error bars represent the ± SD of three biological replicates.

To further investigate the molecular mechanism underlying the accumulation of anthocyanins in response to different light qualities, the transcriptional levels of anthocyanin biosynthetic enzyme genes (*FaPAL*, *FaC4H*, *FaCHS*, *FaCHI1*, *FaF3H*, *FaDFR2*, *FaANS*, and *FaUFGT1*) were detected by RT-qPCR during the light quality treatments. Under dark conditions, these genes showed no significant change in their transcriptional levels (**Figure 2**). Compared to dark and red light, almost all anthocyanin biosynthetic genes showed relatively high transcriptional levels under blue light, especially at 24 h of treatment (**Figure 2**). The expression patterns of these genes can be divided into two categories during blue light irradiation: one is that the expression levels are continuously upregulated during blue light treatment, such as *FaC4H*, *FaCHS*, *FaF3H*, *FaDFR2* and *FaANS*, and the other is that there is a peak expression during the treatment, such as *FaPAL*, *FaCHI1* and *FaUFGT1*. Some anthocyanin biosynthetic genes were also induced in response to red light irradiation, such as *FaCHS*, *FaCHI1* and *FaUFGT1*. Furthermore, the expression of the anthocyanin transport gene, *FaRAP*, was found to be higher under blue light irradiation compared to red light irradiation, similar to the expression pattern observed for the biosynthetic genes (**Figure 2**).

### 3.2 Expression analysis of light signal transduction factors under light irradiation

**Figure 3:**
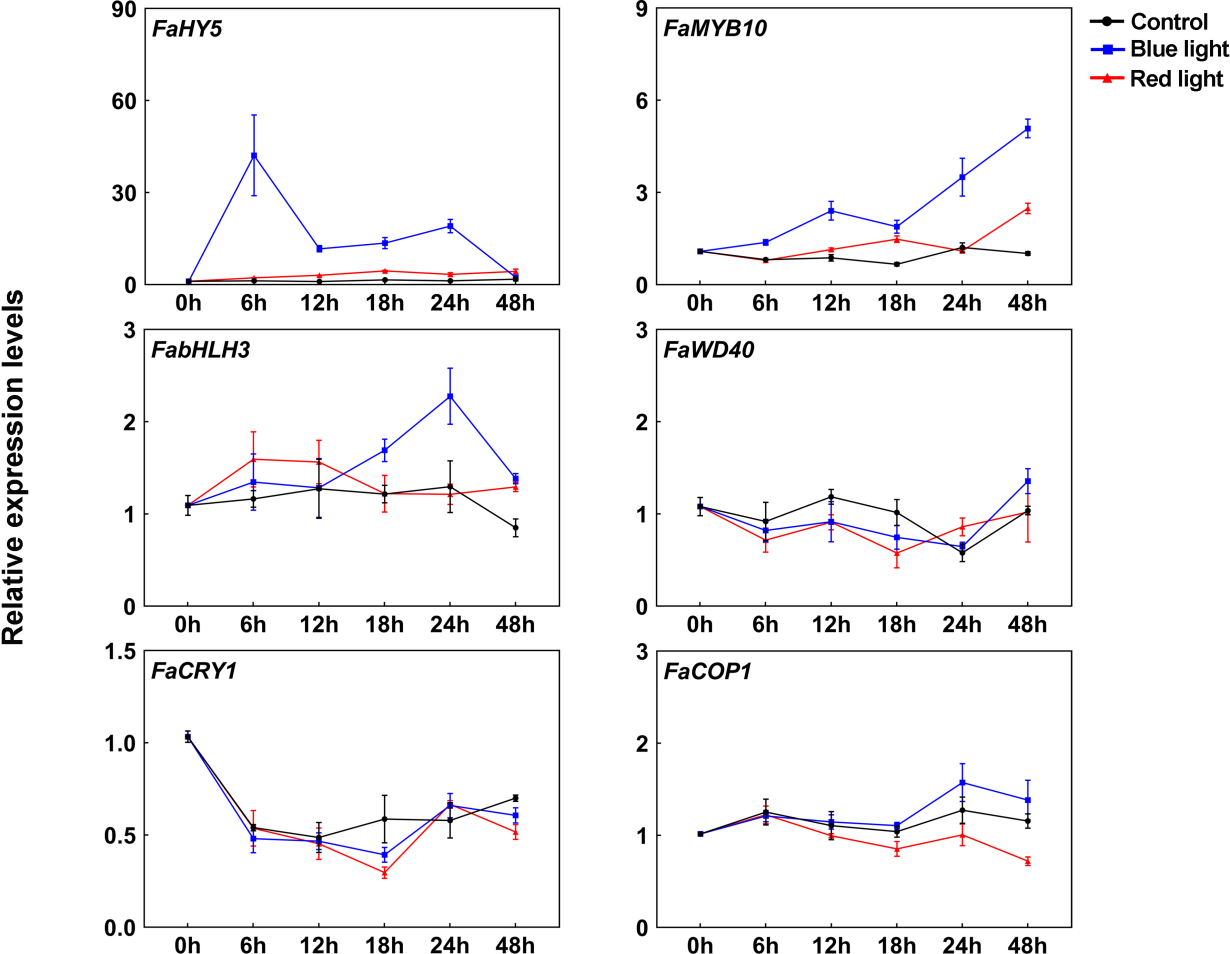
The expression profiles of anthocyanin regulatory genes and light signal transduction factors at different time points. All data were normalized to the transcriptional levels of *FaActin* gene (accession: AB116565.1). Error bars represent the ± SD of three biological replicates.

In strawberry, it has been demonstrated that the expression of anthocyanin structural genes is regulated by MYB10-bHLH3-WD40 (MBW) transcription complex, which plays vital role in anthocyanin accumulation (Schaart et al., 2013). To clarify their functions in blue light-induced anthocyanin accumulation, the transcriptional levels of MBW members were detected. Like other anthocyanin structural genes, *FaMYB10* was induced after light exposure and showed higher transcriptional levels under blue light irradiation than red light irradiation (**Figure 3**). The transcriptional levels of *FabHLH3* was induced by blue light, especially at 24 h of treatment, and there was no significant change in the transcriptional levels of *FabHLH3* under the red light and dark treatment (**Figure 3**). By contrast, the expression levels of *FaWD40* showed little change under the light treatment (**Figure 3**).

Blue light signal transduction pathway has been well identified in model plants, including several important components such as blue light receptor CRYs and downstream signal transduction factors COP1 and HY5 (Ponnu and Hoecker, 2022). To clarify blue light-induced anthocyanin accumulation in strawberry, we cloned *CRY* gene from ‘Benihoppe’ strawberry according to strawberry genome data and named it *FaCRY1*. Then, the amino acid sequences alignment between FaCRY1 and Arabidopsis CRY1 (AtCRY1) was performed. The results showed that both of them contained conserved PHR (photolyase homologous region) domain and CCT (CRY C-terminal extension) domain, which indicates that FaCRY1 and AtCRY1 have functional similarity (**Supplementary Figure 1**). Meanwhile, we also cloned the *COP1* and *HY5* gene and named them *FaCOP1* and *FaHY5*, respectively. RT-qPCR analysis showed that the transcriptional levels of *FaCRY1* were not induced by blue light irradiation but decreased after 0 h among the three tested conditions (**Figure 3**). The expression of *FaCOP1* did not showed obvious change during all treatments (**Figure 3**). Compared to red light irradiation, *FaHY5* exhibited strong response to blue light-induced expression, and its transcriptional levels increased sharply within the first 6 h and then decreased under blue light treatments (**Figure 3**).

**Figure 4:**
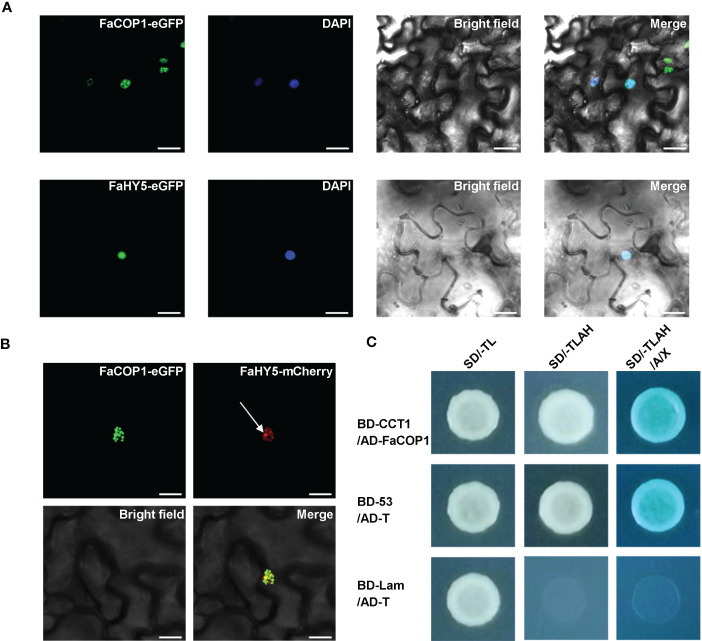
The interaction of blue signal transduction components in strawberry. **(A)** Subcellular localization of the FaCOP1 and FaHY5 protein. The fusion constructs (FaCOP1-eGFP, FaHY5-eGFP) were introduced into *N*. *benthamiana* leaves, and the nucleus was labeled with 4,6-Diamidino-2-phenylindole (DAPI). Bars, 10 μm. **(B)** Recruitment of FaHY5 into FaCOP1 nuclear speckles in living tobacco cell. The arrow points to the red fluorescent speckles of FaHY5-mCherry. Bars, 10 μm. **(C)** The physical interaction of FaCOP1 and CCT1 (COOH- terminus of FaCRY1) was analyzed in yeast cell. Yeast cells that co-transformed pGBKT7-53 (BD-53) and pGADT7-T (AD-T) were used as the positive control. The negative control contained an pGBKT7-Lam (BD-Lam) and AD-T. Co-transformed yeast cells were grown under blue light (50 μmol·m^-2^·s^-1^). SD/-TL indicates SD/-Trp-Leu medium, SD/-TLAH indicates SD/-Trp-Leu-Ade-His medium. SD/-TLAH/A/X indicates SD/-Trp-Leu-Ade-His medium is supplemented with Aureobasidin A (100 ng/mL) and X-α-gal.

In Arabidopsis, it has been shown that AtCRY1 directly interacts with AtCOP1, thereby reducing AtCOP1 E3 ligase activity and relieving the inhibitory effect of AtCOP1 on AtHY5 (Wang et al., 2001; Yang et al., 2001; Holtkotte et al., 2017). To examine whether direct protein-protein interaction between FaCRY1 and FaCOP1, a yeast-two hybrid assay was conducted. The findings indicated that FaCOP1 interacted with the COOH- terminus (amino acids 479-673) of FaCRY1, which has been reported to be an important region of AtCRY1 for the interaction with AtCOP1 in Arabidopsis (**Figure 4C**). In addition, we observed the subcellular localization patterns of FaCOP1 and FaHY5 by transiently expression in *N*. *benthamiana* leaves. As shown in **Figure 4A**, FaCOP1-eGFP (green fluorescent signal) displayed punctate green fluorescent speckles in the nucleus. Unlike FaCOP1-eGFP, FaHY5-eGFP exhibited a uniformly bright green fluorescent signal in the nucleus. Furthermore, when FaHY5-mCherry (red fluorescent signal) was co-expressed with FaCOP1-eGFP in *N*. *benthamiana* leaves, the punctate red fluorescent speckles were observed throughout the uniform red fluorescent background (**Figure 4B**). The alterations in nuclear localization patterns of FaHY5 demonstrated that a possible interaction between FaCOP1 and FaHY5 in living plant cells, which consistent with AtCOP1 and AtHY5 in Arabidopsis (Ang et al., 1998). These results indicated that FaCRY1-FaCOP1-FaHY5 was the functional orthologous of AtCRY1-AtCOP1-AtHY5 and may participates in blue light signal transduction pathway in strawberry.

### 3.3 *FaCOP1* and *FaHY5* restored hypocotyl length and anthocyanin content in arabidopsis mutants under blue light

To further evaluate the roles of *FaCOP1* and *FaHY5* in the light signal transduction pathway, we performed the functional complementation analysis by transforming *FaCOP1* and *FaHY5* into Arabidopsis *cop1* and *hy5* mutants, respectively. At least 3 independent transgenic lines were obtained for each construct and confirmed by semi-quantitative RT-PCR (**Supplementary Figure 2**).

**Figure 5:**
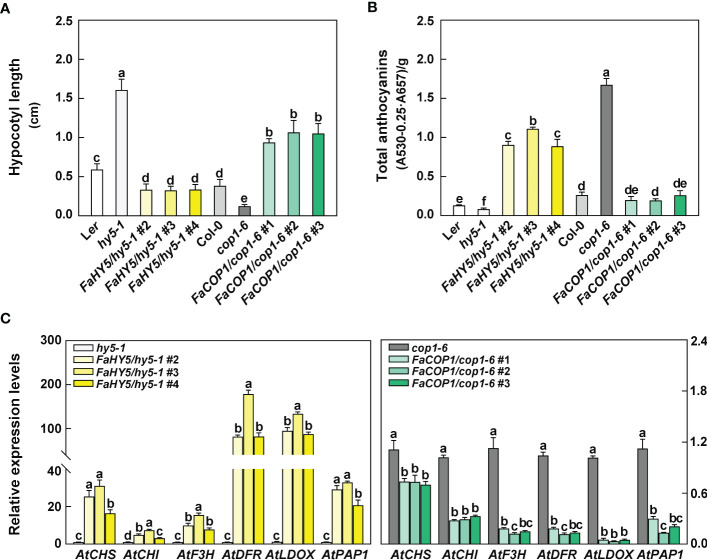
The effect of blue light treatment on hypocotyl length **(A)**, anthocyanin content **(B)** and transcriptional levels of anthocyanin-related genes **(C)** of wild-type, *hy5-1* mutant, *cop1-1* mutant, *FaHY5/hy5-1*, and *FaCOP1*/*cop1-6* transgenic plants. Arabidopsis seedlings were grown in a growth cabinet at 23°C with a 16 h blue-light/8 h dark photoperiod. The 10-day-old seedlings were collected and analyzed. *AtPP2A* were used as the reference gene in Arabidopsis. Error bars represent the ± SD of three biological replicates. Different letters above the bars indicate significantly different values (*p* < 0.05) according to a Least Significant Difference (LSD) test.

The seeds of Arabidopsis, including wild-type (WT), *hy5* mutant, *cop1* mutant and transgenic lines (*FaHY5*/*hy5-1*, *FaCOP1*/*cop1-6*), were sown in the soil in a growth cabinet at 23 °C with a photoperiod of 8-h dark and 16-h blue light. After 10 days of seedlings grown, there was an obvious difference in the length of the Arabidopsis hypocotyl. As shown in **Figure 5A**, among all seedlings, *hy5* mutants exhibited the longest hypocotyl length, which were about 2.7-fold longer than that of the WT (L*er*). *cop1* mutant exhibited the shortest hypocotyl length, which was about 0.3-fold longer than that of the WT (Col-0). The overexpression of *FaHY5* in *hy5* mutant resulted in a shortened hypocotyl (0.25-0.46 cm). In contrast to *FaHY5*, the overexpression of *FaCOP1* promoted the elongation of hypocotyl (0.87-1.30 cm). Moreover, we also measured the total anthocyanin content. Compared to the corresponding mutants, overexpression of *FaHY5* significantly promoted anthocyanin accumulation, whereas overexpression of *FaCOP1* significantly decreased the content of anthocyanin (**Figure 5B**). Likewise, RT-qPCR analysis revealed that the transcriptional levels of anthocyanin structural genes (*AtCHS*, *AtCHI*, *AtF3H*, *AtDFR* and *AtLDOX*) and regulatory gene (*AtPAP1*) in transgenic lines were consistent with the anthocyanin content (**Figure 5C**). All these anthocyanin-related genes generally were up-regulated by *FaHY5* and down-regulated by *FaCOP1*. On the other hand, we investigated the morphology of 10-day-old Arabidopsis seedlings grown in soil under total darkness. As shown in **Supplementary Figure 3**, there was no obvious difference in hypocotyl length among wild-type (L*e*r), *hy5-1* mutant, and transgenic *hy5-1* lines (*FaHY5*/*hy5-1* #2, #3, #4), and their cotyledons were closed. Dark-grown *cop1-6* mutants exhibited light-grown characteristics, including the shortened hypocotyl and expanded cotyledon, while wild-type (Col-0) showed closed cotyledon and lengthened hypocotyl. Meanwhile, we found that transgenic *cop1-6* lines (*FaCOP1*/*cop1-6* #1, #2, #3) showed expanded cotyledon and lengthened hypocotyl. In addition, anthocyanins content were not detected in all Arabidopsis seedlings. Above results indicated that *FaHY5* and *FaCOP1* in strawberry are functional orthologous of *AtHY5* and *AtCOP1* in Arabidopsis, respectively, and their function are affected by light conditions.

### 3.4 *FaHY5* required cofactor to activate the expression of target gene

**Figure 6:**
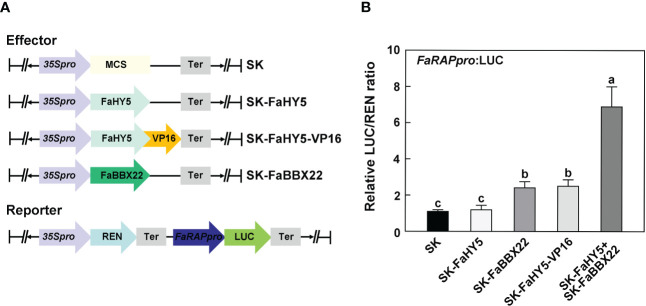
FaHY5 needs co-operative partner to active *FaRAP*’s promoter. **(A)** Schematic diagrams of the effector and reporter constructs used for dual-luciferase assay. SK indicates pGreen II 62-SK vector. **(B)** The dual-luciferase assays showing that FaHY5 required the transactivation domain from VP16 or FaBBX22 to active the expression of LUC. Error bars represent the ± SD of four biological replicates. Different letters above the bars indicate significantly different values (*p* < 0.05) according to a Tamhane’s T2 test.

In Arabidopsis, it has been characterized that AtHY5 lacks a transcriptional activation domain and requires co-operative partners for targets’ activation (Burko et al., 2020; Bursch et al., 2020). To further investigate the mechanism of FaHY5 in the transcriptional regulation of anthocyanin-related genes, we performed the dual-luciferase assay in *N*. *benthamiana* leaves. A 2 kb promoter region of *FaRAP* was subcloned into the upstream of luciferase (named reporter construct). As shown in **Figure 6B**, co-expression of the reporter construct with the effector construct (*35S*:*FaHY5*) did not significantly increase the transcriptional activity of *FaRAP*’s promoter, indicating that FaHY5 alone was unable to induce *FaRAP* expression in *N*. *benthamiana*. We further generated chimeric FaHY5 variants (*FaHY5*-*VP16*) by adding a transcriptional activation domain from VP16 to the C-terminus of FaHY5 protein, which mimicked the activator form of FaHY5 (Dalrymple et al., 1985). When the reporter construct and the effector construct (*35S*:*FaHY5-VP16*) were co-expressed, significantly greater luciferase activities were observed, indicating that FaHY5-VP16 was capable of activating the transcription of *FaRAP*. In our previous work, FaBBX22 has been described as an interacting protein of FaHY5, and FaHY5 promoted the accumulation of anthocyanins depended on FaHY5-FaBBX22 heterodimers in strawberry fruits (Liu et al., 2022). To further test that FaBBX22 is required for FaHY5 to activate *FaRAP* expression, the *Agrobacteria* harboring effector constructs *35S*:*FaHY5* and *35S*:*FaBBX22* were co-infiltrated with reporter construct into *N*. *benthamiana* leaves. Consistent with our previous results in strawberry fruits, FaBBX22 provided the transcriptional activation activity for FaHY5 to increase luciferase activities (**Figure 6B**), which provided the evidence of the important role played by the cofactor in the regulation of downstream gene expression by FaHY5.

### 3.5 Transcriptome analysis in *FaHY5*- and *FaBBX22*-overexpression lines

**Figure 7:**
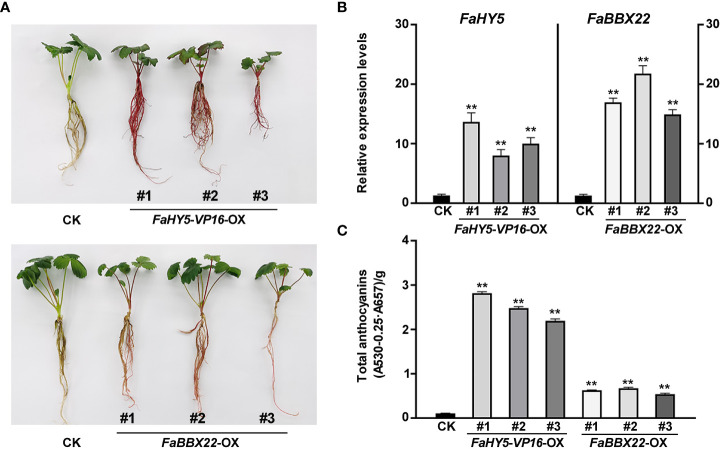
Overexpression of *FaHY5*-*VP16* and *FaBBX22* in ‘Benihoppe’ strawberry. **(A)** Comparison of color of tissue culture seedlings (4-week-old) in control strawberry and transgenic lines. CK, control samples (wild-type ‘Benihoppe’ strawberry); *FaHY5*-*VP16-*OX, *FaHY5*-*VP16*’s overexpression lines; *FaBBX22*-OX, *FaBBX22*’s overexpression lines. **(B)** The relative expression levels of *FaHY5* and *FaBBX22* in corresponding transgenic lines. The data were normalized using the CK samples. **(C)** Total anthocyanin content of CK and transgenic lines. Asterisks indicate significant differences compare with CK (two-tailed Student’s *t*-test, **p < 0.01).

All previous studies showed that HY5 works mainly as a transcriptional activator in the regulation of anthocyanin biosynthesis (An et al., 2017; Liu et al., 2018; Bai et al., 2019b). To further investigate the effects of FaHY5 on anthocyanin accumulation in strawberry, *FaHY5*-*VP16* (the activator form of FaHY5) under *35S* promoter (pCAMBIA1301-*35S*:*FaHY5-VP16*) was transformed into the strawberry cultivar ‘Benihoppe’ and 3 independent transgenic (*FaHY5*-*VP16-*OX) lines were obtained (**Supplementary Figure 4**). The observation of the appearance of transgenic plants showed that the *FaHY5*-*VP16-*OX plants were redder than wild-type ‘Benihoppe’ strawberry (**Figure 7A**). Furthermore, we measured the anthocyanin content of wild-type (control samples, CK) and transgenic strawberry samples. The results showed that the total anthocyanin content of *FaHY5*-*VP16-*OX lines were about 24 times higher than that of CK, suggesting that the activator form of FaHY5 could promote the accumulation of anthocyanins in strawberry (**Figure 7C**). Additionally, we also obtained transgenic strawberry plants that overexpressed the *FaBBX22* gene. Similar to our previous results in transgenic calli, the *FaBBX22*-overexpression (*FaBBX22*-OX) strawberry plants accumulated more anthocyanin than CK, which further confirmed the positive role of *FaBBX22* in anthocyanin accumulation (**Figures 7A**, **C**).

**Figure 8:**
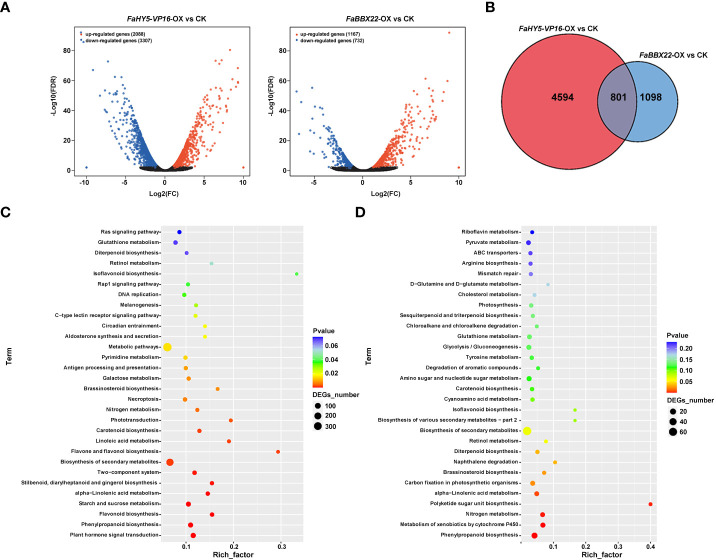
Transcriptome analysis of *FaHY5*-*VP16-*OX and *FaBBX22*-OX strawberry. **(A)** The volcano plot of the DEGs between transgenic strawberry and CK. **(B)** The Venn graph illustrates the comparison of the numbers of differentially expressed genes between transgenic strawberries and the control group. **(C)** The enrichment analysis of KEGG pathway in the differentially expressed genes between *FaHY5*-*VP16-*OX strawberry and CK. **(D)** The enrichment analysis of KEGG pathway in the differentially expressed genes between *FaBBX22*-OX strawberry and CK.

To gain insight into the effects of *FaHY5*-*VP16-*OX and *FaBBX22*-OX on the transcriptional levels of downstream genes, transgenic lines (*FaHY5*-*VP16-*OX #1 and *FaBBX22*-OX #2) with the highest expression levels of target gene and wild-type ‘Benihoppe’ strawberry plants were subjected to RNA-seq analysis. Over 98.86 million raw reads were generated in the nine libraries (three replicates each for the CK and transgenic lines) and approximate 94% reads were mapped to *F*. ananassa genome (**Supplementary Table 2**). The edgeR software (R3.6.3) was used to identify the differentially expressed genes (DEGs). Compared to the CK, 5395 DEGs and 1899 DEGs were identified in *FaHY5*-*VP16-*OX samples and *FaBBX22*-OX samples, respectively, using the Q value (adjusted *p* value) ≤ 0.05 as the standard. Among these DEGs, 2088 genes were up-regulated, and 3307 genes were down-regulated in *FaHY5*-*VP16-*OX samples, and 1167 genes were up-regulated and 732 genes were down-regulated in *FaBBX22*-OX samples (**Figure 8A** **;** **Supplementary Table 3** **;** **Supplementary Table 4**). We also found that 801 genes were co-regulated by *FaHY5*-*VP16-* and *FaBBX22*-overexpression (**Figure 8B**; **Supplementary Table 5**). To determine the biological interpretation of DEGs, the KEGG analysis were performed, and the 30 most enriched pathways are shown in **Figure 8C**. In *FaHY5*-*VP16-*OX *vs.* CK, “plant hormone signal transduction”, “phenylpropanoid biosynthesis” and “flavonoid biosynthesis” were the first three terms with significant enrichment. In *FaBBX22*-OX *vs.* CK, the first three significantly enriched terms were “phenylpropanoid biosynthesis”, “metabolism of xenobiotics by cytochrome P450” and “nitrogen metabolism” (**Figure 8D**). Additionally, there were other biological pathways, such as biosynthesis of secondary metabolites, brassinosteroid biosynthesis and carotenoid biosynthesis, *etc*., also showed high *p*-value in the KEGG analysis (**Figures 8C**, **D**). These results further confirmed that *FaHY5* and *FaBBX22* were involved in the secondary metabolites and other pathways as well.

## 4 Discussion

### 4.1 Blue light rapidly induced anthocyanin accumulation in strawberry fruits by up-regulating the expression of anthocyanin-related genes

Anthocyanins, the predominant pigment in ripe strawberry fruits, not only give fruits a brilliant red color, but also benefit the nutritional value of fruits (Castillejo et al., 2020). Light is an important environment factor affecting the anthocyanin accumulation (Ma et al., 2021). Plants have evolved sophisticated light signal transduction systems to cope with different light intensity, quality, duration and direction (Lau and Deng, 2012; Liang et al., 2019). In particular, it has been shown that light of different wavelengths has distinct impacts on anthocyanin accumulation in fruits (Jaakola, 2013). In this study, we investigated the effect of different light environments on anthocyanin accumulation in ‘Benihoppe’ strawberries. Our results showed that constant blue-light irradiation, rather than red-light irradiation, can stimulate rapid and significant anthocyanin accumulation in post-harvest fruits at 10°C (**Figure 1**). Similarly, the highest concentration of anthocynains was detected in blue-light irradiated strawberry fruits treated at 23°C with a 12 h light/12 h dark photoperiod (Kadomura-Ishikawa et al., 2013). The rapid accumulation was due to the relatively high transcriptional levels of anthocyanin structural and regulatory genes under blue light irradiation (**Figures 2****,** **3**). Meanwhile, we found that the response speed and response time of these anthocyanin-related genes to light treatment were different, which may be related to the regulation of their promoters by light. In strawberry, single structural gene is sufficient to control anthocyanin accumulation. For instance, Hoffmann et al. (2006) obtained completely white strawberry fruit by RNAi-mediated *FaCHS* silencing. Recently studies have demonstrated that anthocyanin transporter *rap* (reduced anthocyanins in petioles) mutants failed to accumulate anthocyanin in strawberry fruits and petioles (Luo et al., 2018; Gao et al., 2020). As shown in **Figure 2**, several anthocyanin structural genes (*FaCHS*, *FaCHI1*, and *FaUFGT1*) were also upregulated under red light exposure, but did not induce anthocyanin accumulation, which might be attributed to: (1) the transcriptional abundance of these structural genes was insufficient to induce anthocyanin accumulation; (2) the anthocyanin accumulation depends on the activation of most structural genes in its biosynthesis pathway. The molecular mechanism of differentially regulating anthocyanin accumulation by red and blue light remains to be further investigated in strawberry.

### 4.2 The blue light signaling pathway is conserved in strawberry and model plants

In plants, the blue light signal transduction pathways have been established, including blue light receptors, light signal repressors, and light-regulated TFs (Ponnu and Hoecker, 2022). Blue light is sensed by photoreceptor CRYs which subsequently initiate the light signal transduction pathway by inhibiting the COP1-mediated protein degradation. We isolated the Arabidopsis *AtCRY1* homologous gene (*FaCRY1*) from strawberry and identified its basic domains. The results showed that FaCRY1 consist of two domains: the N-terminal Photolyase Homologous Region (PHR) domain and the CRY C-terminal extension (CCT) domain (**Supplementary Figure 1**). In Arabidopsis, the PHR domain of CRYs is responsible for binding the primary chromophore, flavin adenine dinucleotide (FAD), which plays the key role in light absorption (Yang et al., 2017). Under blue light, the CCT domain plays a critical role in mediating protein-protein interactions between CRYs and signaling proteins (Wang et al., 2001). E3 ubiquitin ligase COP1 acts as the negative regulator of light signaling by ubiquitinating and degrading the light-regulated TFs in darkness (Lau and Deng, 2012). One of these TFs, HY5 is considered to be a signal hub acting downstream of photoreceptors and promotes photomorphogenesis (Gangappa and Botto, 2016). The Arabidopsis *hy5* mutants display longer hypocotyl and have a decrease in anthocyanin content under all light conditions, while *cop1* mutants show the opposite phenotypes (Deng et al., 1991; Deng et al., 1992; Oyama et al., 1997; Ang et al., 1998). At the transcriptional level, *FaCRY1* expression in strawberry fruits was not induced by light exposure (**Figure 3**), which is similar to Arabidopsis *AtCRY1* (Ahmad and Cashmore, 1993). However, Tóth et al. (2001) revealed that circadian clock regulates *AtCRY1* expression, and the promoter activity of *AtCRY1* was 2- to 3-fold higher in the light than in the dark. In eggplant, the expression levels of *SmCRY1* in the peel of normally irradiated eggplant were higher than that of bagged eggplant (Jiang et al., 2016). Compared to darkness, light quality treatments had no significant effect on *PpCRY1* transcript abundance in pear (Tao et al., 2018). This difference may be due to the different experimental setup, such as developmental stages, sampling times, and detection methods, or differential regulatory mechanisms between species. Moreover, the transcript abundances of *FaCOP1* were relatively stable during our light treatments. In addition to the transcriptional level, whether *FaCRY1* and *FaCOP1* are regulated by light at the post-transcriptional level remains to be confirmed.

In our work, yeast two-hybrid results revealed that the COOH- terminus of FaCRY1, containing the CCT domain, physically interacted with FaCOP1 under blue light (**Figure 4**). The protein-protein interaction is the same as in Arabidopsis, apple, and pear (Li et al., 2013; Holtkotte et al., 2017; Tao et al., 2018). In Arabidopsis, the CCT domain of AtCRY1 directly interacts with AtCOP1, thereby suppressing AtCOP1 activity and promoting the accumulation of AtCOP1-targeted proteins under blue light (Wang et al., 2001; Holtkotte et al., 2017). It has previously been demonstrated that AtCOP1 can recruit HY5 into nuclear speckles by a direct interaction (Ang et al., 1998). We observed that FaCOP1-eGFP localized to distinct speckles in the nuclei of tobacco mesophyll cell, similar to the localization of AtCOP1 in nuclear speckles (Ang et al., 1998). Meanwhile, FaHY5-mCherry was found to colocalize with FaCOP1-eGFP in these nuclear speckles, which was different from the uniform nuclear localization shown by FaHY5-eGFP alone (**Figure 4**). These results suggest that the interaction of CRY-COP1-HY5 in blue light signal transduction pathway is conserved in strawberry. We also identified the functions of FaHY5 and FaCOP1 in corresponding Arabidopsis mutants (**Figure 5** **;** **Supplementary Figure 3**). The hypocotyl length and anthocyanin content of transgenic Arabidopsis seedlings were restored by *FaHY5*- and *FaCOP1*-overexpression. Our results demonstrate that the biological functions of FaHY5 and FaCOP1 are conserved and that further studies are needed to verify the regulatory mechanisms between them. Kadomura-Ishikawa et al. (2013) found that another blue-light photoreceptor, FaPHOT2, had a positive effect on anthocyanin accumulation in strawberry fruits, as demonstrated through transient gene expression assays under blue light. Taken together, there are at least two pathways in strawberries that respond to blue light and regulate anthocyanin accumulation. One pathway involves the FaCRY-FaCOP1-FaHY5 signal transduction module, while the other is mediated by the FaPHTO2 photoreceptor.

### 4.3 FaHY5 needs other cofactors to activate anthocyanin accumulation

The Arabidopsis basic leucine zipper TF HY5 regulates multiple biological processes, including seedling photomorphogenesis, pigment accumulation, chloroplast development and nutrient assimilation (Gangappa and Botto, 2016). The homologs of Arabidopsis HY5 have also been characterized to be involved in the regulation of light-induced anthocyanin accumulation in several horticultural plants (Loyola et al., 2016; An et al., 2017; Liu et al., 2018; Zhao et al., 2021). For example, blue light irradiation can promote the accumulation of SlHY5 both at the transcription and post-translation level, thereby increasing anthocyanins’ content in tomato (Liu et al., 2018). Similarly, the transcriptional level of *FaHY5* from strawberry was induced sharply within 6 h after blue light exposure (**Figure 3**). Therefore, FaHY5 may play an important role in blue light signaling pathway. In Arabidopsis, RNA-seq and ChIP-seq experiments found that HY5 can bind to thousands of genes and regulate their transcription (Lee et al., 2007; Zhang et al., 2011). HY5 targets include anthocyanin structural and regulatory genes, such as *CHS*, *F3H*, *DFR*, *UFGT*, *MYB75*. The complication is that HY5 lacks the transactivation domain and fails to activate downstream genes’ expression alone (Burko et al., 2020). We also found that FaHY5 did not have a transactivation activity in yeast cell (**Supplementary Figure 5**). In addition, we provided in planta evidences that FaHY5 had activator activity depending on the interacting partner, such as the B-box TF FaBBX22 (**Figure 6**). These results suggest that strawberry FaHY5 needs other cofactors to function, which is similar to previous reports in Arabidopsis and pear (Bai et al., 2019b; Burko et al., 2020). However, Zhao et al. (2021) reported that overexpression of *HY5* from peach alone significantly increased the promoter activity of its own and several anthocyanin-related genes, suggesting HY5 proteins may show different characteristics in peach and strawberry, two closely related species.

So far, some B-box TFs have been identified as the interacting protein of HY5, which are involved in the regulation of anthocyanin accumulation, such as AtBBX20, AtBBX21, AtBBX22, and AtBBX24 in Arabidopsis, PpBBX16, PpBBX18, and PpBBX21 in pear, MdBBX20 and MdBBX21 in apple (Job et al., 2018; Bai et al., 2019a; Bai et al., 2019b; Fang et al., 2019; Bursch et al., 2020; Zhang et al., 2021). In our previous work, we identified 51 B-box family members in cultivated strawberry and verified the function of FaBBX22 (Ye et al., 2021; Liu et al., 2022). As a transcriptional activator, FaBBX22 physically interacted with FaHY5 to form the FaBBX22/FaHY5 complex, thereby promoting anthocyanin accumulation in strawberry (Liu et al., 2022). In present work, we obtained transgenic strawberry plants that overexpressed the *FaBBX22* and *FaHY5*-*VP16* (chimeric activator form of FaHY5), and coloration assays showed that both *FaBBX22* and *FaHY5*-*VP16* increased anthocyanins’ content (**Figure 7**). RNA-seq analysis of transgenic plants indicated that overexpression of *FaHY5*-*VP16* induced changes in the expression of more than 5000 genes, of which 801 overlapped with samples from plants overexpressing *FaBBX22* (**Figure 8** **;** **Supplementary Table 4**). These genes co-regulated by FaHY5 and FaBBX22 may play an important role in the specific biological process regulated by FaHY5/FaBBX22 complex. On the other hand, 4594 genes and 1098 genes were identified as DEGs specifically in *FaHY5*-*VP16-*OX and *FaBBX22*-OX samples, respectively (**Figure 8**). Our results identified a series of downstream genes regulated by FaHY5 and FaBBX22, but the biological significance of their differential expression across different samples still needs to be clarified by further studies.

## Data availability statement

The transcriptome data from this study have been uploaded to NCBI Sequence Read Archive database under BioProject ID: PRJNA916624.

## Author contributions

Conceptualization and funding acquisition: HT and YQL. Methodology: LT, YPW, LZ, SX, and XW. Software: YQL and WH. Validation: YQL, YTZ, ML, and YXL. Writing—original draft preparation and project administration: YQL. Writing-editing: YW, XRW, YQZ, YL, and QC. The published version of the manuscript has been reviewed and approved by all authors. All authors contributed to the article.
