# Supplementary material for: The blue light signal transduction module FaCRY1-FaCOP1-FaHY5 regulates anthocyanin accumulation in cultivated strawberry
Source: Front Plant Sci. 2023 Jun 9;14:1144273. doi: 10.3389/fpls.2023.1144273 (PMC10289005; doi:10.3389/fpls.2023.1144273)
Supplement: Supplementary file 1 [file DataSheet_1.docx]

Supplementary Material

The Blue Light Signal Transduction Pathway Is Involved in Anthocyanin Accumulation in Cultivated Strawberry

Haoru Tang*, Yongqiang Liu, Li Tang, Yiping Wang

*** Correspondence:** Haoru Tang: htang@sicau.edu.cn


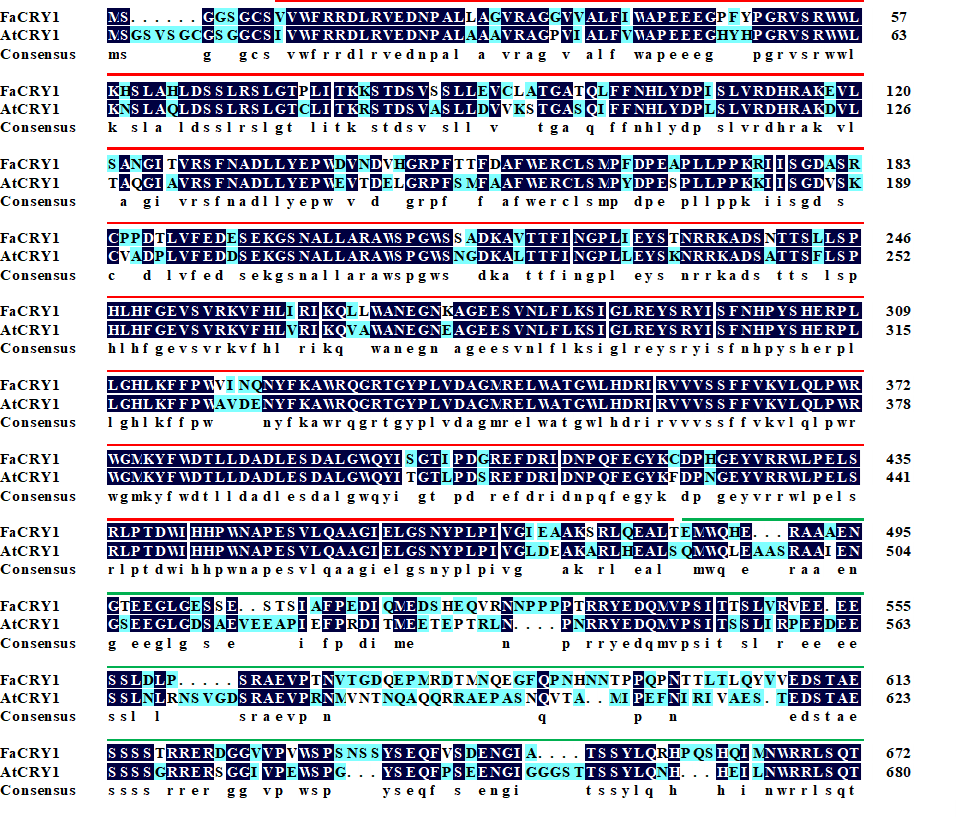


**Supplementary Figure 1.** The amino acid sequences alignment between FaCRY1 and AtCRY1. The red line region indicates the PHR domain and the green line region indicates the CCT domain.

**
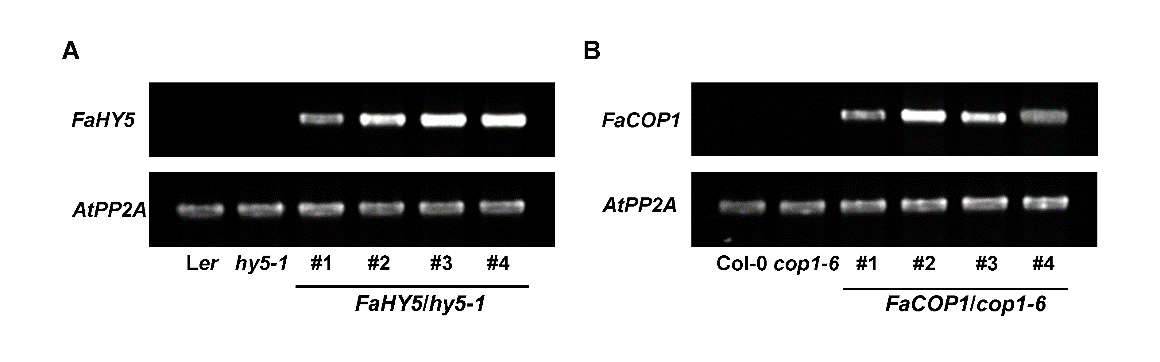
**

**Supplementary Figure 2.** The *cop1* mutant (*cop1-6*) and *hy5* mutant (*hy5-1*) transformed with *FaCOP1* and *FaHY5*, respectively. At least 3 independent transgenic lines were obtained for each construct and confirmed by semi-quantitative RT-PCR. Transgenic line #2, #3, #4 in *FaHY5*/*hy5-1* and line #1, #2, #3 in *FaCOP*1/*cop1-6* were chosen for phenotype analysis.


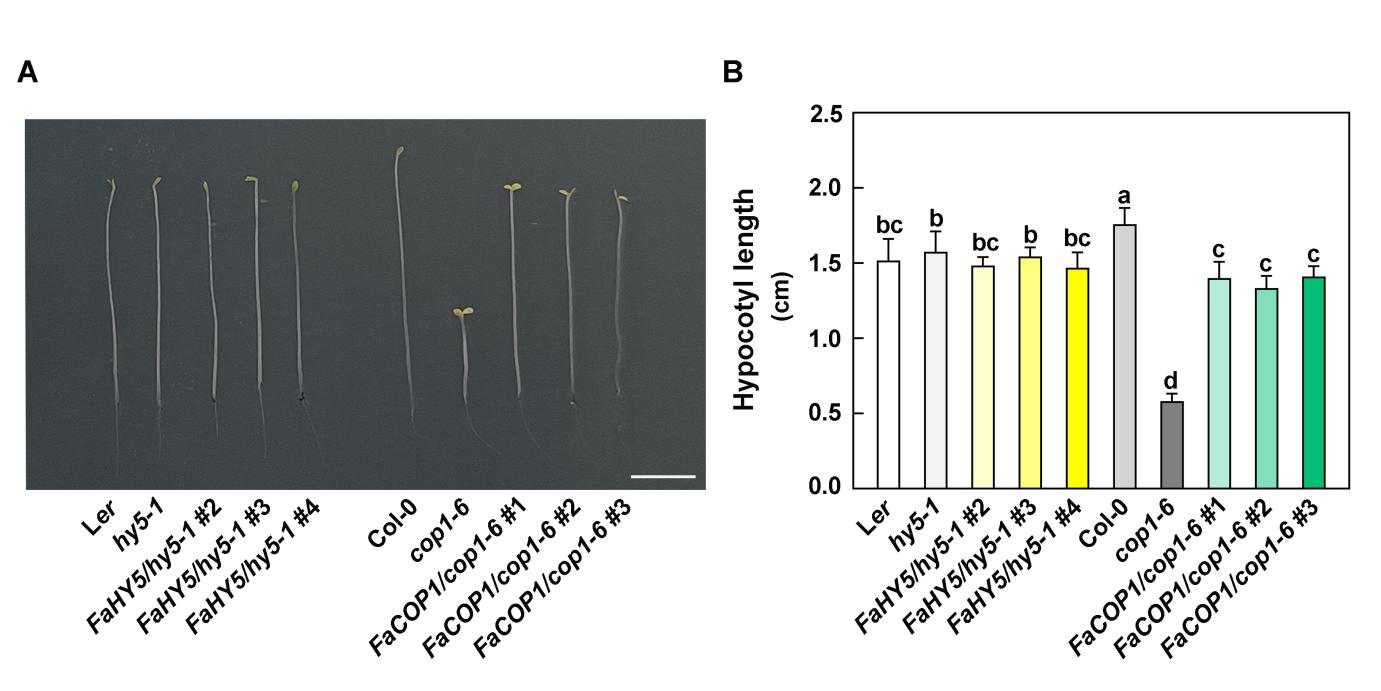


**Supplementary Figure 3.** Effects of the overexpression of *FaHY5* and *FaCOP1* in Arabidopsis under dark treatment. (A) Phenotypes of wild-type, mutants (*hy5-1*, *cop1-6*), and transgenic Arabidopsis (*FaHY5*/*hy5-1*, *FaCOP1*/*cop1-6*) grown at 23 ℃ in total darkness for 10 days. Scale bars, 0.5 cm. (B) Hypocotyl length of Arabidopsis seedlings grown at 23 ℃ in total darkness for 10 days. Error bars represent the ± SD of three biological replicates. Different letters above the bars indicate significantly different values (*p* < 0.05) according to a Least Significant Difference (LSD) test.

**
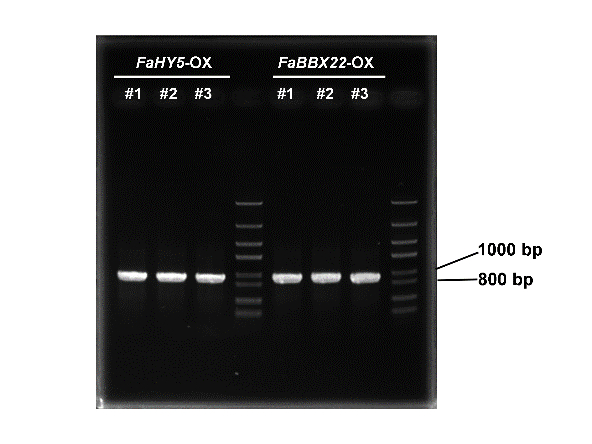
**

**Supplementary Figure 4.** Detection of hygromycin resistance gene (*HygR*) in transgenic strawberry lines by PCR.

**
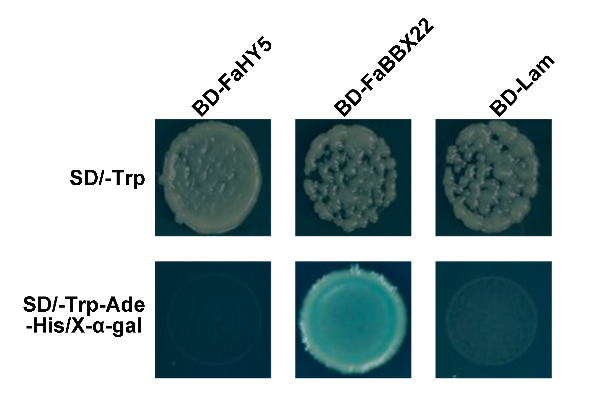
**

**Supplementary Figure 5.** Transcriptional activity analysis of FaHY5 in yeast cell. The yeast cells transformed with pGBKT7-Lam (BD-Lam) vector were used as a negative control, and the yeast cells transformed with pGBKT7-FaBBX22 (BD-FaBBX22) vector were used as a positive control.
